# A sulfur-free peptide mimic of surfactant protein B (B-YL) exhibits high in vitro and in vivo surface activities

**DOI:** 10.12688/gatesopenres.12799.2

**Published:** 2018-07-10

**Authors:** Frans J. Walther, Monik Gupta, Larry M. Gordon, Alan J. Waring

**Affiliations:** 1 Department of Pediatrics, Los Angeles Biomedical Research Institute at Harbor-UCLA Medical Center, Torrance, CA, 90502, USA

**Keywords:** Synthetic lung surfactant, Surfactant Protein B (SP-B), SP-B peptide mimics, Respiratory Distress Syndrome (RDS), Circular Dichroism (CD) spectroscopy, Fourier-Transform InfraRed (FTIR) spectrometry, Captive Bubble Surfactometry (CBS), lung lavage rabbit model

## Abstract

**Background**: Animal-derived surfactants containing surfactant proteins B (SP-B) and C (SP-C) are used to treat respiratory distress syndrome (RDS) in preterm infants. SP-B (79 residues) plays a pivotal role in lung function and the design of synthetic lung surfactant. Super Mini-B (SMB), a 41-residue peptide based on the N- and C-domains of SP-B covalently joined with a turn and two disulfides, folds as an α-helix hairpin mimicking the properties of these domains in SP-B. Here, we studied ‘B-YL’, a 41-residue SMB variant that has its four cysteine and two methionine residues replaced by tyrosine and leucine, respectively, to test whether these hydrophobic substitutions produce a surface-active, α-helix hairpin.

**Methods:** Structure and function of B-YL and SMB in surfactant lipids were compared with CD and FTIR spectroscopy, and surface activity with captive bubble surfactometry and in lavaged, surfactant-deficient adult rabbits.

**Results:** CD and FTIR spectroscopy of B-YL in surfactant lipids showed secondary structures compatible with peptide folding as an α-helix hairpin, similar to SMB in lipids. B-YL in surfactant lipids demonstrated excellent *in vitro* surface activity and good oxygenation and dynamic compliance in lavaged, surfactant-deficient adult rabbits, suggesting that the four tyrosine substitutions are an effective replacement for the disulfide-reinforced helix-turn of SMB. Here, the B-YL fold may be stabilized by a core of clustered tyrosines linking the N- and C-helices through non-covalent interactions involving aromatic rings.

**Conclusions:** ‘Sulfur-free’ B-YL forms an amphipathic helix-hairpin in surfactant liposomes with high surface activity and is functionally similar to SMB and native SP-B. The removal of the cysteines makes B-YL more feasible to scale up production for clinical application. B-YL’s possible resistance against free oxygen radical damage to methionines by substitutions with leucine provides an extra edge over SMB in the treatment of respiratory failure in preterm infants with RDS.

## Introduction

Lung surfactant is a lipid-protein mixture that is synthesized by alveolar type II cells and secreted into the alveolus where it reduces surface tension at the air-liquid interface. Mammalian lung surfactant harvested by lavage consists of approximately 80% phospholipids, 10% neutral lipids and 10% protein ^1^. Phospatidylcholine (PC), and particularly dipalmitoylphosphatidylcholine (DPPC), is the major phospholipid constituent of lung surfactant. DPPC enhances the formation of a rigid film at the air-liquid interface that reduces alveolar surface tension to low values during dynamic compression, whereas fluid phospholipids and neutral lipids are important because they significantly improve film spreading ^2^,^3^. The highly hydrophobic surfactant protein B (SP-B) and, to a lesser extent, surfactant protein C (SP-C), facilitate the absorption of phospholipids into the air-liquid interface and thus play an important role in the reduction of alveolar surface tension. SP-B is pivotal for normal lung function, by hereditary SP-B deficiency being fatal in newborn infants ^4^ and also in SP-B knockout mice ^5^. Human SP-B is a 79 amino-acid, lipid-associating monomer (MW 8.7 kDa) found in the lung as a covalently linked homodimer. Early theoretical studies based on homology comparisons indicated that the SP-B monomer consists of 4-5 α-helices ^6^,^10^ with three intramolecular disulfide bridges (i.e., Cys-8 to Cys-77, Cys-11 to Cys-71 and Cys-35 to Cys-46) ^11^, and belongs to the saposin protein superfamily ^12^. The helical bundle for SP-B folds into two leaves, with one leaf having α-helices 1 (N-terminal helix), 5 (C-terminal helix) and 4 and the other composed of α-helices 2 and 3 ^13^,^14^.

**Figure 1.**
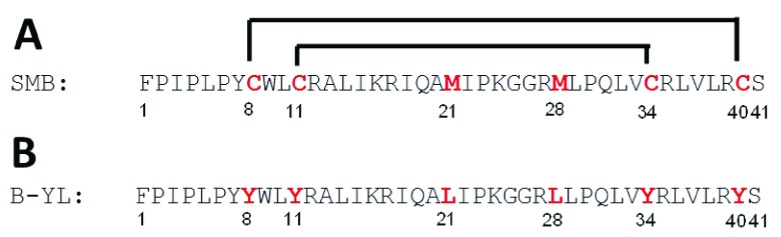
Sequences for Super Mini-B (SMB) and B-YL. ( **A**) SMB (41 amino-acid residues; 1-letter amino-acid notation), with the N- and C-terminal Phe-1 and Ser-41 as indicated, as well as the sulfur-containing cysteines (Cys-8, Cys-11, Cys-34, Cys-40) and methionines (Met-21, Met-28) in red. The disulfide-linkages are shown between Cys-11 and Cys-34 and between Cys-8 and Cys-40. ( **B**) B-YL (41 residues) shares the same sequence as its parent SMB, except that the cysteines and methionines are replaced by tyrosines (Tyr-8, Tyr-11, Tyr-34, Tyr-40) and leucines (Leu-21, Leu-28) in red, respectively.

Intratracheal administration of animal-derived lung surfactants, which contain only polar lipids and native SP-B and SP-C, has greatly improved morbidity and mortality of premature infants with neonatal respiratory distress syndrome (RDS) as a result of surfactant-deficiency due to lung immaturity ^15^. Existing clinically available formulations are extracted from lung lavages or homogenates from pigs (Curosurf®) and cows (Infasurf®, Survanta®), and contain small amounts of SP-B and SP-C (<< 2% of total weight) in a lipid extract with DPPC as its main component. Based on the predicted 3D-saposin motif for SP-B, we have developed minimal SP-B constructs that have desirable structural properties and maintain high activities in animal models of surfactant deficiencies ^9^,^10^. For example, Super Mini-B (SMB) is a 41-residue, ‘short-cut’ peptide ( Figure 1A), based on the primary sequence, secondary structure and tertiary folding of the known sequence of native SP-B (79-residues), that mimics the high surfactant activity of its parent protein ^10^,^14^,^16^. SMB incorporates the N-terminal α-helix ( residues 8-25) and C-terminal α-helix ( residues 63-78) of native SP-B as a single linear peptide ( Figure 1A), joined together with a customized turn to form a α-helix hairpin (α-helix/turn/α-helix, αtα) ^17^. SMB has two vicinal disulfide bonds (i.e., Cys-8 to Cys-77 and Cys-11 to Cys-71) that further covalently link the N- and C-terminal α-helices, and also a hydrophobic N-terminal insertion sequence (i.e., residues 1-7; FPIPLPY). Experimental procedures validated the above structural model for SMB, including conventional C-FTIR spectroscopy, mass spectroscopy, I-TASSER, ModWeb and SWISS MODEL homology modeling, and Molecular Dynamics (MD) simulations in lipid mimics and lipid bilayers ^14^,^16^,^18^. When formulated with a lipid composition that mimics that of native lung surfactant, SMB has shown excellent surface activity with fresh and stored preparations, which was closely associated with the formation of an α-helix hairpin ^10^,^14^,^18^.

Because surfactant therapy is life-saving in preventing and treating RDS in preterm infants, on-going research is studying whether surfactant therapy may be efficaciously extended to pediatric and adult patients with clinical acute lung injury (ALI) or the acute respiratory distress syndrome (ARDS) ^19^. ALI and ARDS may each be caused by direct exposure of lungs to pathogens, oxidative air pollutants, cigarette smoke and other irritants in the alveolar space, and by the presence of endogenous reactive oxygen species (ROS) in damaged lungs due to permeability edema or the inflammatory response. Subsequent oxidative alterations may produce dysfunctional and even inactive lung surfactant in these diseases ^20^,^21^. SP-B is an important target for ROS-induced oxidative surfactant inactivation ^22^,^23^. Specifically, oxidation of native SP-B involves alterations in the methionines (Met-29, Met-65) and tryptophan (Trp-9), which correlates well with the loss of *in vitro* surfactant activity ^23^. Mimics based on the native SP-B sequence may be likewise sensitive to oxidation processes. Kim *et al.* ^24^ reported that ozone treatment of SP-B(1–25), an SP-B mimic whose sequence overlaps residues 1–25 of SMB ( Figure 1A) and native SP-B, variably oxidized amino-acids known to react with ozone. In contrast to the nearly complete homogenous oxidation of the susceptible SP-B(1–25) residues (i.e., Cys-8, Cys-11, Trp-9, and Met-21) in the solvent phase, only a limited subset of residues (Trp-9 and Met-21) oxidized in the hydrophobic interfacial environment provided by the lipid surfactant layer ^24^. In additional studies, Hemming *et al.* ^25^ showed that exposure of either SP-B _1–25_ or SMB at the air-water interface to dilute ozone ( 2 ppm) produced a rapid loss of surface activity (i.e., increase in surface tension). Because decreases in tryptophan fluorescence occurred concurrently with increasing surface tension for these two SP-B mimics ^25^, it is likely that oxidative disruption of the indole ring of Trp-9 can play a role in the diminished surface activity in the full-length protein, possibly due to a fraying of the N-terminal α-helix ^25^,^26^.

Synthetic lung surfactant with SP-B and SP-C peptide mimics offers substantial advantages over current animal-derived surfactants for treating surfactant deficiency in neonatal RDS. Current research on synthetic lung surfactant has focused on designing peptide mimics of natural surfactant proteins that are highly effective, stable, and easy to manufacture ^9^,^10^. Here, we conducted structural and functional experiments on ‘B-YL’ ( Figure 1B), a 41-residue SMB variant that has its four Cys and two Met residues replaced by Tyr (Tyr-8, Tyr-11, Tyr-34 and Tyr-40) and Leu (Leu-21 and Leu-28), respectively, and tested whether these hydrophobic substitutions produce a surface-active, α-helix hairpin. Tyrosine was substituted for cysteine because of its aromatic ring interactions that emulate disulfide formation ^27^,^29^ and methionine, that is easily oxidized, was replaced by leucine based on its similar molecular structure and polarity ^30^.

## Methods

### Materials

HPLC grade chloroform, methanol, trifluoroethanol (TFE), and acetonitrile were purchased from Fisher Scientific (Pittsburgh, PA 15275), trifluoroacetic acid from Sigma Chemical Co (Saint Louis, MO 63103), NMR quality deuterated water was from Aldrich Chemical Co. (St. Louis, MO 63103), and Sephadex LH-20 chromatography gel from Pharmacia (Uppsala, Sweden). Phospholipids were supplied by Avanti Polar Lipids (Alabaster, AL 35007), and Sodium Dodecyl Sulfate (SDS) detergent was from Sigma Chemical Co (Saint Louis, MO 63103).

The Super Mini-B (SMB) peptide sequence ( Figure 1A) was synthesized using a standard Fmoc protocol with a Symphony Multiple Peptide Synthesizer (Protein Technologies, Inc., Tucson, AZ 87514) or a CEM Liberty microwave synthesizer (CEM Corporation, Mathews, NC 28104), cleaved-deprotected and purified using reverse phase HPLC as described earlier ^14^,^18^. This synthesis protocol included folding of the peptide in a structure-promoting TFE-buffer solvent system to promote oxygen-mediated disulfide linkages between Cys-8 and Cys-40 and between Cys-11 and Cys-34 ^10^,^14^. This covalently stabilized connectivity gave the peptide a helix-hairpin conformation, comparable to the topological organization seen for the N- and C-terminal helical domains of the saposin family of proteins ^9^,^10^,^12^. The synthesis of B-YL was identical to that of SMB, except for replacing cysteines with tyrosines (Tyr-8, Tyr-11, Tyr-34, and Tyr-40) and methionines with leucines (Leu-21 and Leu-28), and also omitting the oxidation step. The purified SMB and B-YL peptides were each freeze-dried directly, and the masses were confirmed by MALDI TOF mass spectrometry as described previously ^17^. Peptide concentrations were routinely quantitated using UV absorbance based on the assay procedure developed by Anthis and Clore ^31^.

### Preparation of proteins and lipids in surfactant dispersions

Peptide and lipids were formulated as lipid-peptide dispersions to have a total of 3% by mole fraction of SMB or B-YL and 35 mg of total lipid [i.e., DPPC: POPC: POPG 5:3:2 mole:mole:mole] per mL of dispersion ^18^. The peptide was dissolved in 10 mL of trifluoroethanol and co-solvated with the lipid in chloroform, followed by removal of the solvents with a stream of nitrogen gas and freeze drying of the resulting lipid-peptide film to remove residual solvent. The film was then dispersed with Phosphate Buffered Saline and the sample flask containing the hydrated film was rotated for 1 h at 60°C to produce a solution of multilamellar vesicles (MLVs) ^14^. Lipid controls were similarly prepared but without peptide. These dispersions were then stored at 4°C prior to structural and functional measurements. To determine the molecular mass of peptides formulated with lipids, the peptide was separated from lipid using normal phase chromatography with Sephadex LH-20 ^32^. Mass spectral analysis of the B-YL peptide indicated there was no change in the molecular weight due to oxidation of tyrosines or tryptophan for one year when formulated with surfactant lipids.

### Circular dichroism (CD) spectroscopy of the secondary structure of the B-YL mimic

CD spectra (190–260 nm) of the B-YL peptide in various structure-promoting environments, including surfactant dispersions, were measured with a JASCO 715 spectropolarimeter (Jasco Inc., Easton MD 21601). The instrument was routinely calibrated for wavelength and optical rotation using 10-camphorsulphonic acid ^33^. The sample solutions were scanned using 0.01 cm pathlength cells at a rate of 20 nm per minute, a sample interval of 1 nm, and a temperature of 37°C. Sample concentration was determined by UV absorbance at 280 nm ^31^. Peptide concentration was 100 μM in sample solutions with either TFE:Phosphate buffer (10 mM, pH 7.0) having a volume ratio of 4:6 (v/v), SDS micelles (100 mM) in phosphate buffer (10 mM, pH 7.0), or Single Unilamellar Vesicles (SUVs) of simulated surfactant lipids (DPPC: POPC: POPG; 5:3:2, mole:mole:mole). Surfactant lipid SUVs were prepared at a concentration of 2.6 μM lipids/mL of phosphate buffer solution (10 mM, pH 7.0) by bath sonication for 10 minutes ( https://avantilipids.com/tech-support/liposome-preparation/). Sample spectra were baseline corrected by subtracting spectra of protein-free solution from that of the protein-bound solution and expressed as the Mean Residue Ellipticity [θ] _MRE_ as shown in equation (1):

$${[\text{θ]}}_{\text{MRE}}=([\text{θ]}\times \text{100})/(1\times \text{c}\times \text{N})\;(1)$$

The symbol θ is the measured ellipticity in millidegrees, l is the pathlength in cm, N is the number of residues in the peptide, and c is the concentration of the peptide in mM.

Quantitative estimates of the secondary structural contributions were also made with SELCON 3 ^34^ using the spectral basis set for membrane proteins, option 4 implemented from the DichroWeb website ^35^,^36^.

### Attenuated-Total-Reflectance Fourier-transform infrared (ATR-FTIR) spectrometry of the B-YL and SMB peptides

ATR-FTIR spectra were recorded at 37°C using a Bruker Vector 22 FTIR spectrometer (Pike Technologies, Fitchburg, WI 53719) with a deuterium triglyceride sulfate (DTGS) detector. The spectra were averaged over 256 scans at a gain of 4 and a resolution of 2 cm ^14^. For spectra of B-YL and SMB in TFE solutions, self-films were first prepared by air-drying peptide originally in 100% HFIP onto a 50 × 20 × 2 mm, 45° attenuated total reflectance (ATR) crystal for the Bruker spectrometer. The dried peptide self-films were then overlaid with solutions containing 40% TFE/60% deuterated-10 mM sodium phosphate buffer (pH 7.4), at a peptide concentration of 470 μM. Control solvent samples were similarly prepared for FTIR analysis, but without peptide. Spectra of peptides in solvent were obtained by subtraction of the solvent spectrum from that of peptide solvent. For FTIR spectra of B-YL and SMB in either SDS micelles or surfactant lipids, each lipid-peptide solution was transferred onto a germanium ATR crystal. The aqueous solvent was then removed by flowing nitrogen gas over the sample to produce a thick lipid-peptide (lipid:peptide ratios of 10:1, mole:mole) ^14^. The multilayer film was then hydrated to ≥35% with deuterated water vapor in nitrogen for 1 h before acquiring the spectra ^37^. The spectra for either the B-YL or SMB peptides in the film were obtained by subtracting the spectrum of a peptide-free control sample from that of the peptide-bound sample. The relative amounts of α-helix, β-turn, β-sheet, or random (disordered) structures in lipid-peptide films were estimated using Fourier deconvolution (GRAMS AI 8, version 8.0, Thermo Fisher Scientific, Waltham, MA 02451). The respective areas of component peaks were calculated using curve-fitting software ( Igor Pro, version 1.6, Wavemetrics, Lake Oswego, OR 97035) ^38^. FTIR frequency limits were: α-helix (1662-1650 cm ), β-sheet (1637-1613 cm ), turn/bend (1682-1662 cm ), and disordered or random (1650-1637 cm ) ^39^.

### Captive bubble surfactometry

Adsorption and surface tension lowering ability of surfactant preparations were measured with a captive bubble surfactometer at physiological cycling rate, area compression, temperature, and humidity ^14^. The captive bubble surfactometer used here was a fully-computerized version of that described and built by Schürch and coworkers ^40^,^41^. Quasi-static compression and expansion of the air bubble was performed in discrete steps at a rate of 5% of the bubble volume every 10 sec with continuous video recording of the bubble shape. Dynamic compression and expansion cycling was performed between 10 and 110% of the original bubble area at a cycling rate of 20 cycles/min. Both modalities show extreme flattening of the air bubble in active surfactant preparations. We used a B-YL surfactant mixture consisting of 3 mole% of B-YL peptide formulated in surfactant lipids (DPPC:POPC:POPG 5:3:2, mole:mole:mole) with a concentration of 35 mg/mL. Surfactant lipids alone were used as negative control and SMB surfactant (3 mole% of SMB in surfactant lipids) and the clinical surfactant Curosurf® (porcine lung extract containing both SP-B and SP-C and 80 mg/mL of lipids) as positive control. We routinely analyze surfactant preparations at an average surfactant lipid concentration of ~25 μg/mL in the bubble chamber, but as Curosurf® is more concentrated than synthetic surfactant, we applied 1 µL of synthetic surfactant at 35 mg/mL and 0.5 µL of Curosurf® at 80 mg/mL to the bubble chamber (~1.5 mL volume), and performed all measurements in quadruplicate.

### *In vivo* experiments

Animal experiments were performed under established protocols reviewed and approved by the Institutional Animal Care and Use Committee of the Los Angeles Biomedical Research Institute at Harbor-UCLA Medical Center (LA BioMed protocol # 020645). All procedures and anesthesia were in accordance with the American Veterinary Medical Association (AMVA) guidelines. Any suffering of the rabbits was ameliorated by providing optimal anesthesia and sedation as outlined below.

The lung lavage rabbit model represents a relatively pure state of surfactant deficiency over at least 6–8 h and allows for serial measures of arterial blood gases and lung compliance in ventilated, surfactant-deficient animals with a clinical picture of respiratory failure as seen in neonatal RDS and ALI/ARDS. Respiratory failure secondary to surfactant deficiency has a high mortality if not treated with a highly surface-active surfactant preparation. Thirty-three young adult, New Zealand white rabbits, weighing 1.0–1.4 kg, were purchased from IFPS Inc. (Norco, CA). The animals were housed as pairs for a minimum of 24 h in the C.W. Steers Biological Resources Center of LA BioMed, using large cages with non-traumatic and moisture absorbent bedding, and provided with rabbit toys and food and water *ad libitum*. Husbandry was provided by veterinary technicians under supervision of a veterinarian. The number of animals has been determined from a population correlation=0.6, α=0.05, tails=2, and power=0.8, which gives a sample size of 16 (2x8). Therefore, we generally use 8 animals to test clinical efficacy of an experimental surfactant preparation (here: 3 mole% B-YL in surfactant lipids, n=9) with groups of 8 animals as positive controls (here: Curosurf® and 3 mole% SMB in surfactant lipids, both n=8) and 8 animals for negative controls (here: surfactant lipids alone [DPPC:POPC:POPG 5:3:2 mole:mole:mole], n=8). These treatment group sizes allow significant differences to be found between rabbits receiving an optimal surfactant and positive and negative controls. Animals were assigned to a surfactant preparation using a randomized algorithm and experiments were performed in a special laboratory area set up for the provision of intensive care.

The rabbits received anesthesia with 50 mg/kg of ketamine and 5 mg/kg of acepromazine intramuscularly prior to placement of a venous line via a marginal ear vein. After intravenous administration of 2 mg/kg of propofol and 2 mg/kg of midazolam for anesthesia and sedation, a small incision in the skin of the anterior neck allowed for placement of an endotracheal tube and a carotid arterial line. After insertion of the endotracheal tube, mechanical ventilation was initiated and muscle paralysis induced with intravenous vecuronium (0.1 mg/kg) to prevent spontaneous breathing. During the ensuing duration of mechanical ventilation, anesthesia consisted of continuous intravenous administration of 30 mg/kg/h of propofol and, as needed, additional intravenous dosages of 2 mg/kg of midazolam for sedation, whereas muscle paralysis was maintained with hourly intravenous administration of 0.1 mg/kg of vecuronium. Maintenance fluid was provided with a continuous infusion of lactated Ringer’s solution at a rate of 10 mL/kg/h. Heart rate, arterial blood pressures and rectal temperature were monitored continuously (Labchart® Pro, ADInstruments Inc., Colorado Springs, CO).

The rabbits were ventilated with a volume-controlled rodent ventilator (Harvard Apparatus, South Natick, MA) using a tidal volume 7.5 mL/kg, a positive end-expiratory pressure of 3 cm H _2_O, an inspiratory/expiratory ratio of 1:2, 100% oxygen, and a respiratory rate sufficient to maintain the partial pressure of carbon dioxide (PaCO _2_) at 40 mmHg. Airway flow and pressures and tidal volume were monitored with a pneumotachograph connected to the endotracheal tube (Hans Rudolph Inc., Kansas City, MO). When the partial pressure of oxygen in arterial blood (PaO _2_) was >500 mmHg at a peak inspiratory pressure <15 cm H _2_O in 100% oxygen, surfactant deficiency was induced with repeated intratracheal instillation and removal of 30 mL/kg of warmed normal saline. When the PaO _2_ was stable at <100 mmHg (average 4 lavages), B-YL surfactant or a surfactant control (SMB, Curosurf® or surfactant lipids alone) was then instilled intratracheally at a dose of 100 mg/kg body weight, similar to dosages given to premature infants with RDS. Curosurf® is more concentrated (80 mg/mL) than SMB and B-YL surfactant and lipids alone (35 mg/mL), so Curosurf® was given at 1.25 mL/kg and SMB and B-YL surfactant and lipids alone at 2.9 mL/kg. Oxygenation was followed by measuring arterial pH and blood gases and lung compliance at 15 min intervals over a 2 h period. Dynamic lung compliance was calculated by dividing tidal volume/kg body weight by changes in airway pressure (peak inspiratory pressure minus positive end-expiratory pressure) (mL/kg/cm H _2_O).

Animals were sacrificed 2 h after surfactant administration with an overdose (200 mg/kg) of intravenous pentobarbital. End-points were oxygenation and dynamic lung compliance at 120 min after surfactant administration.

### Statistical analysis

All data are expressed as mean ± SEM. Statistical analyses (IBM Statistical Package for the Social Sciences (SPSS) 23.0) used Student's t-test for comparisons of discrete data points, and functional data were analyzed by one-way analysis of variance (ANOVA) with Scheffe's post hoc analysis to adjust for multiple comparisons. Differences were considered statistically significant if the P value was <0.05.

## Results

### Spectroscopic analysis of B-YL and SMB in lipid mimic and surfactant lipid environments

**Figure 2.**
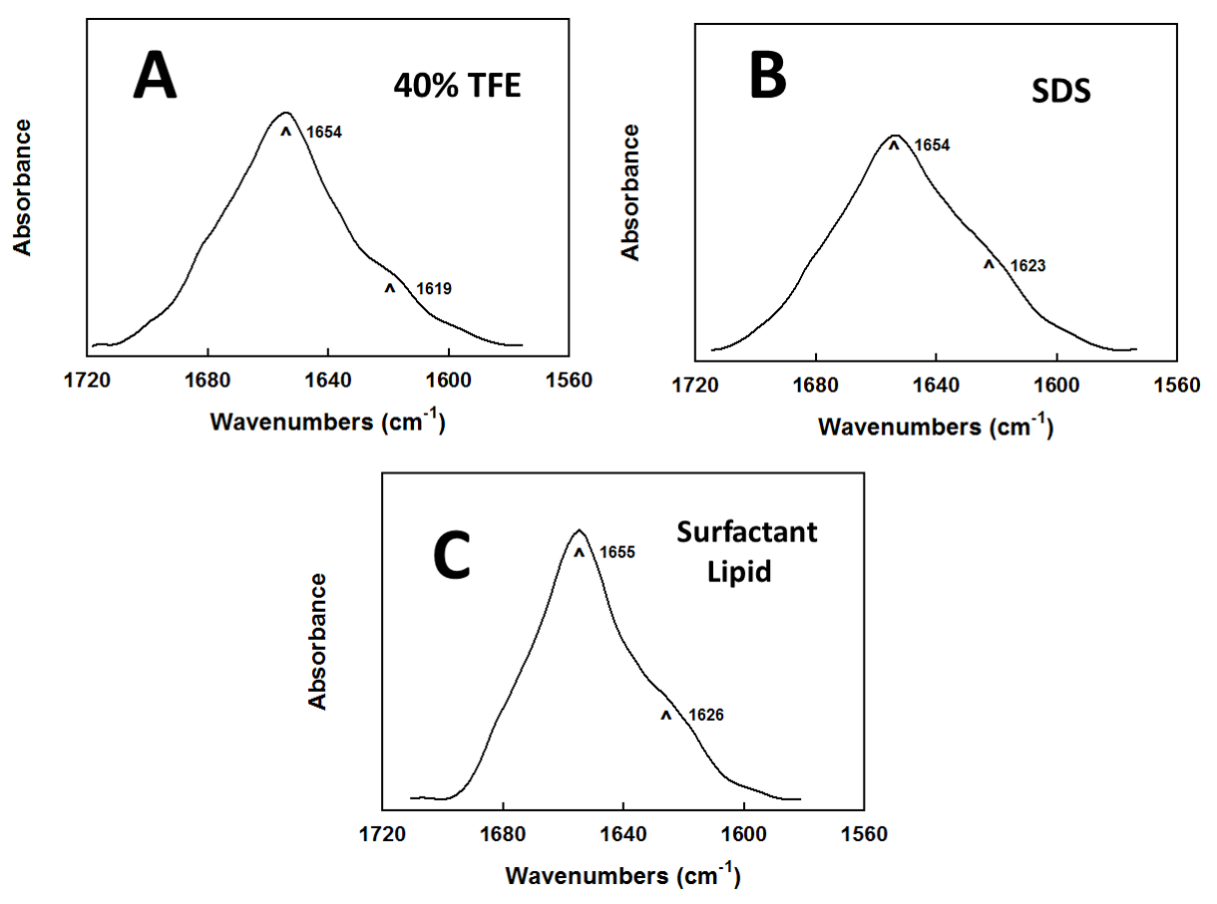
FTIR spectra of the B-YL mimic in several lipid-mimetics and surfactant lipids. Attenuated total reflectance Fourier transform infrared (ATR-FTIR) spectral plots show the absorbance (in arbitrary units) as a function of wavenumbers (cm ) (See **Methods** and **Results**). ( **A**) Deuterated aqueous 40% TFE. ( **B**) Deuterated aqueous SDS. ( **C**) Deuterated aqueous surfactant lipid. In ( **A**), ( **B**) and ( **C**), the IR spectra each show dominant α-helical components centered at 1654–1655 cm (arrows), with minor bands at 1619–1626 cm (arrows) due to β-sheet, 1682–1662 cm due to loop-turn/bend, and 1650–1637 cm due to disordered or random conformations. Peptide concentrations were 470 μM for the TFE solvent spectra, and 10:1 lipid:peptide (mole:mole) for the SDS detergent and surfactant lipid spectra. The areas under each absorbance curve are normalized.

The secondary structures for B-YL in either lipid mimetics (i.e., 40% TFE/60% deuterated-sodium phosphate buffer, pH 7.4 and deuterated aqueous SDS micelles) or surfactant lipids [i.e., deuterated aqueous DPPC: POPC: POPG 5:3:2 (mole:mole:mole) multilayers] were studied with conventional C-FTIR spectroscopy. Representative FTIR spectra of the amide I band for B-YL in these environments were all similar ( Figure 2), each showing a principal component centered at 1654–1655 cm with a small low-field shoulder at 1619–1626 cm . Because earlier FTIR investigations of proteins and peptides ^39^,^42^ have assigned bands in the range of 1650–1659 cm as α-helical, while those at 1613–1637 cm are characteristic of β-sheet, B-YL probably assumes α-helical and β-sheet structures and possibly other conformations in these environments. Self-deconvolutions of the Figure 2 spectra confirmed that B-YL is polymorphic, primarily adopting α-helix but with significant contributions from β-sheet, loop-turn and disordered components ( Table 1). Interestingly, the relative proportions of secondary conformations determined from FTIR spectra of B-YL (i.e., α-helix > loop-turn disordered β-sheet) in both lipid-mimetics and surfactant lipids of varying polarity are all comparable, suggesting an overall stability of the B-YL structure that is remarkably conserved. It is also important to note that the proportions of these secondary conformations are all compatible with B-YL principally assuming an α-helix hairpin ^10^,^14^,^18^.

**Table 1.** Spectroscopic proportions of secondary structure ^a^ for B-YL and SMB in lipid-mimetics and surfactant lipid.

System	% Conformation ^a^			
	α-Helix	Loop-Turn	β-Sheet	Disordered
*FTIR Analysis of B-YL* ^b^				
40% TFE	44.9	20.1	14.0	21.0
SDS	43.5	21.5	15.4	19.6
Surfactant Lipid	44.3	18.9	15.1	21.7
*CD Analysis of B-YL* ^c^				
40% TFE	46.9	11.8	15.1	26.2
SDS	52.0	13.9	9.9	24.2
Surfactant Lipid	44.6	16.3	13.3	25.8
*FTIR Analysis of SMB* ^d^				
40% TFE	47.1	19.4	20.2	13.3
SDS	44.9	22.2	12.2	20.7
Surfactant Lipid	42.3	27.5	11.7	18.5

Tabulated results are means from four closely-reproduced separate determinations for each condition and spectral type.

See Figure 2. ATR-FTIR spectra were estimated for proportions of the secondary structure for B-YL in 40% trifluoroethanol (TFE), sodium dodecyl sulfate SDS micelles and surfactant lipid-MLV films using self-deconvolution of the peptide amide I band (see **Methods** and **Results**).

**Figure 3.**
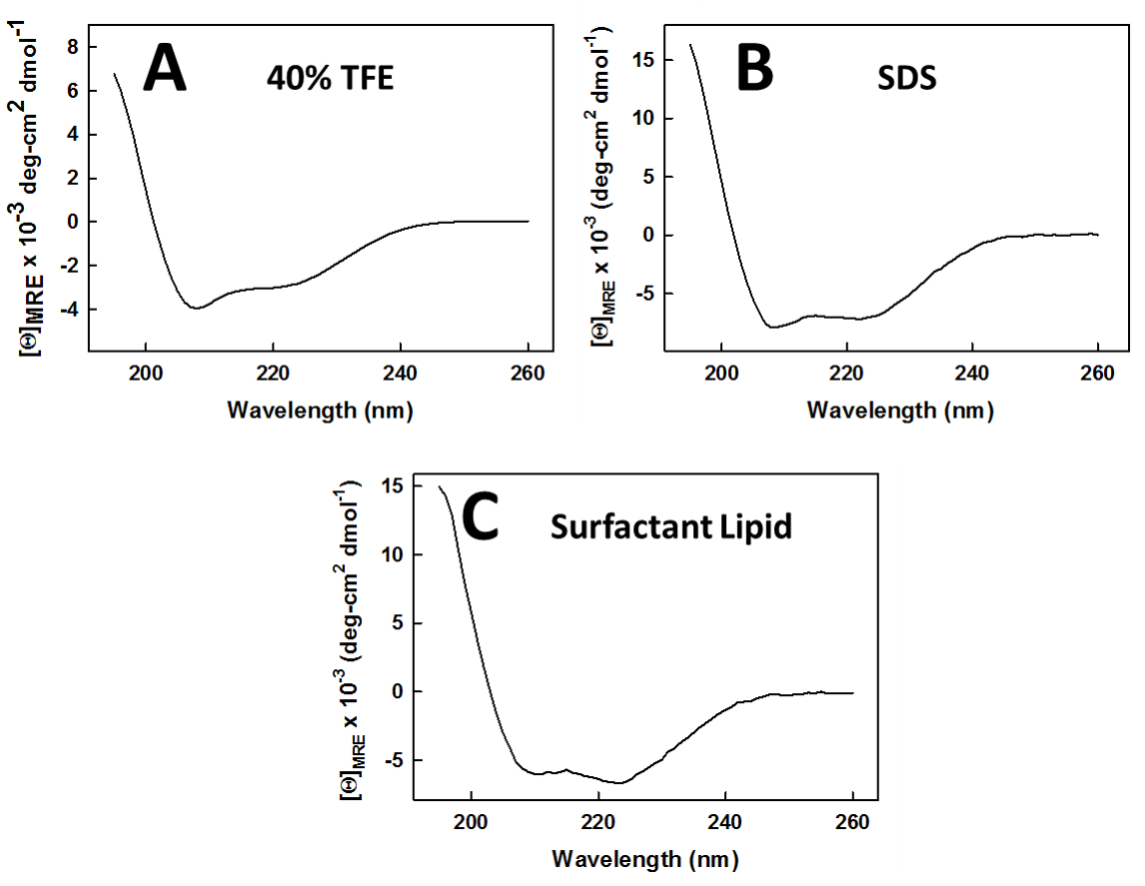
CD spectra of the B-YL mimic for SP-B in several lipid-mimetics and surfactant lipid. Circular Dichroism (CD) spectral plots show the mean residue ellipticities ([θ] _MRE_ x 10 deg-cm dmol ) as a function of wavelength (nm). ( **A**) 40% TFE. ( **B**) SDS. ( **C**) Surfactant Lipid. The double minimum at 208 and 222 nm in each plot indicates that α-helix is the dominant secondary structure for B-YL in these environments. Peptide concentrations were 100 μM. The optical pathlength was 0.01 cm and the temperature was 37°C. Spectra represent the average of 8 scans.

See Figure 3. Circular dichroism (CD) spectra were analyzed for proportions of the secondary structure for the B-YL mimic in 40% TFE, SDS micelles or surfactant lipid using spectral deconvolution (see **Methods** and **Results**).

**Figure 4.**
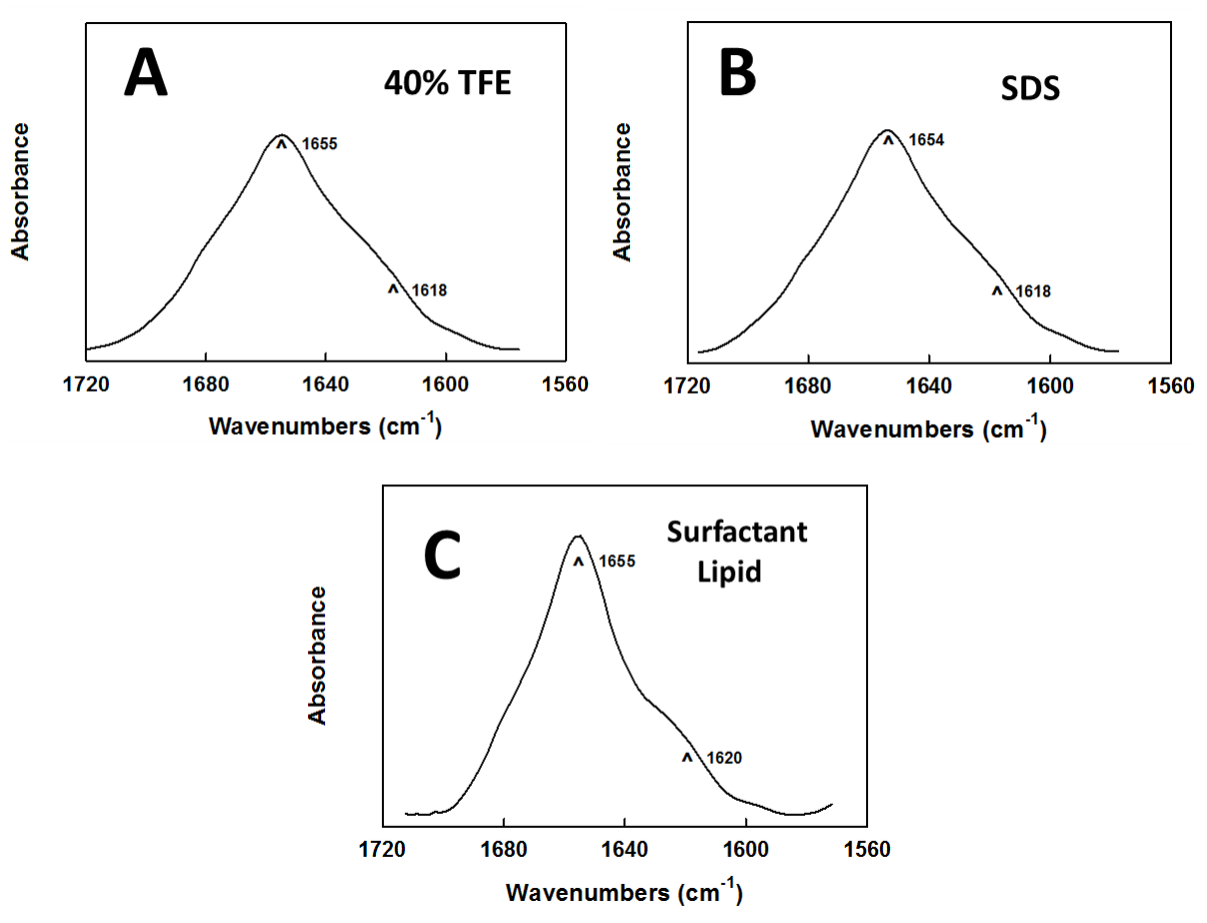
FTIR spectra of Super Mini-B (SMB) in several lipid-mimetics and surfactant lipids. Attenuated total reflectance Fourier transform infrared (ATR-FTIR) spectral plots show the absorbance (in arbitrary units) as a function of wavenumbers (cm ) (See **Methods** and **Results**). ( **A**) Deuterated aqueous 40% TFE. ( **B**) Deuterated aqueous SDS. ( **C**) Deuterated aqueous surfactant lipid. In ( **A**), ( **B**) and ( **C**), the IR spectra each show dominant α-helical components centered at 1654–1655 cm (arrows), with minor bands at 1618–1620 cm (arrows) due to β-sheet, 1682–1662 cm due to loop-turn/bend, and 1650–1637 cm due to disordered or random conformations. Peptide concentrations were 470 μM for the TFE solvent spectra, and 10:1 lipid:peptide (mole:mole) for the SDS detergent and surfactant lipid spectra. The areas under each absorbance curve are normalized.

See Figure 4. ATR-FTIR spectra were estimated for proportions of the secondary structure for Super Mini-B (SMB) in SDS micelles and surfactant lipid-MLV films using self-deconvolution of the peptide amide I band (see **Methods** and **Results**).

The secondary conformations for B-YL in lipid mimics (i.e., 40% TFE, aqueous SDS) or synthetic surfactant lipids [aqueous (DPPC:POPC:POPG 5:3:2 mole:mole:mole] were also studied with Circular Dichroism (CD) spectroscopy, to validate the above FTIR results. CD spectra for B-YL in these environments ( Figure 3) were all similar, each indicating a major α-helical component characterized by a double minimum at 208 and 222 nm ^43^,^45^. Deconvolutions of the Figure 3 spectra showed that B-YL is polymorphic, principally adopting α-helix ( 45–52%), but with significant contributions (i.e., 10–26% each) from loop-turn, disordered/random and β-sheet components ( Table 1). Interestingly, the secondary conformation proportions determined from CD analysis for B-YL (i.e., α-helix > random loop-turn β-sheet) in both surfactant lipids and lipid mimetics are all compatible with B-YL folding as an α-helix hairpin ^18^. Moreover, the overall maintenance of these secondary conformations from CD spectra in Table 1 additionally supports our FTIR findings that B-YL assumes a stable 3D-structure in environments of varying polarity.

Comparative FTIR spectroscopic studies were next performed on Super Mini-B (SMB) in lipid mimics and surfactant lipids to assess whether the B-YL substitutions in Figure 1 perturb the structure of the parent SMB. The secondary structures for SMB in lipid mimetics (i.e., deuterated 40 % TFE, deuterated aqueous SDS) and surfactant lipids (deuterated aqueous DPPC:POPC:POPG 5:3:2 mole:mole:mole) were investigated with conventional C-FTIR spectroscopy. Figure 4 shows that representative FTIR spectra of the amide I band for SMB in these environments were similar, each indicating a dominant α-helical component centered at 1654–1655 cm with a small low-field shoulder due to β-sheet at 1618–1620 cm ^40^,^43^. Self-deconvolution of the FTIR spectra in Figure 4 demonstrated that SMB in either TFE, SDS or surfactant lipids folds with secondary structures that are characteristic of the α-helix hairpin ( Table 1). Our finding that the respective secondary structure profiles for B-YL and SMB are similar in Table 1 suggests that the amino-acid substitutions in B-YL (e.g., four Cys residues replaced by Tyr) do not disrupt the α-helix hairpin conformation.

### Captive bubble surfactometry

**Figure 5.**
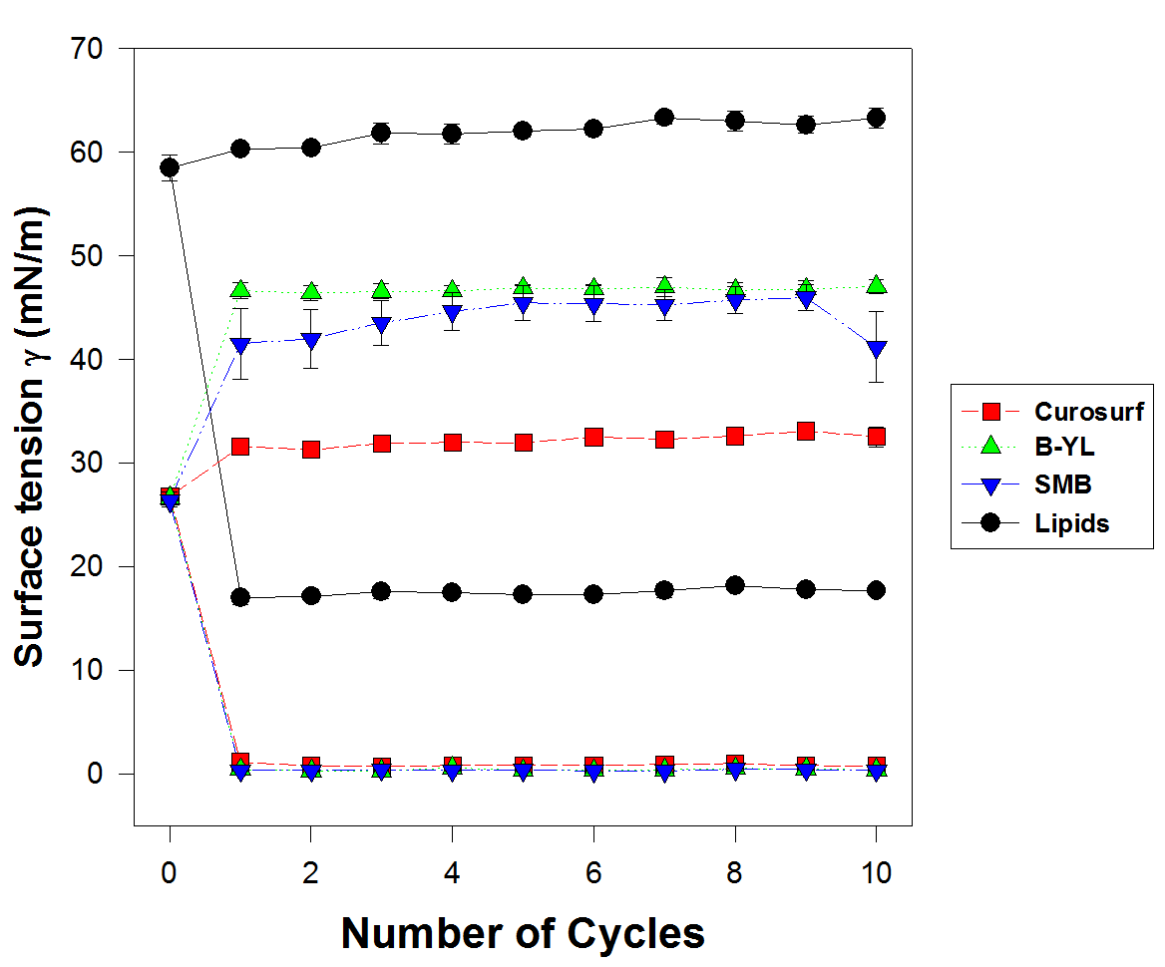
Surface tension reduction activity measured with captive bubble surfactometry. Surface activity of 3 mole% B-YL and Super Mini-B (SMB) in surfactant lipids were compared with a clinical surfactant (Curosurf®) as positive control and surfactant lipids alone (DPPC:POPC:POPG 5:3:2 mole:mole:mole) as negative control. The lower part of each curve indicates minimum surface tension and the upper part indicates maximum surface tension during 10 compression-expansion cycles. Minimum surface tension values of B-YL and SMB surfactant were similar to those of Curosurf®. Values are mean ± SEM of N=4-5.

Captive bubble surfactometry of B-YL and SMB surfactants (3 mole% peptide in DPPC:POPC:POPG 5:3:2 mole:mole:mole), Curosurf®, and surfactant lipids alone demonstrated excellent surface activity of B-YL ( Figure 5). Surface activity of BYL and SMB surfactant and Curosurf® were consistently and equally low during quasi-static cycling with values ≤1 mN/m, indicating that the modifications in the B-YL peptide did not lead to a loss in *in vitro* surface activity compared to its parent peptide SMB. In contrast, minimum surface tension values of surfactant lipids alone far exceeded those of B-YL, SMB and Curosurf® surfactant and amounted to 18 mN/m (p<0.001).

### *In vivo* experiments

**Figure 6.**
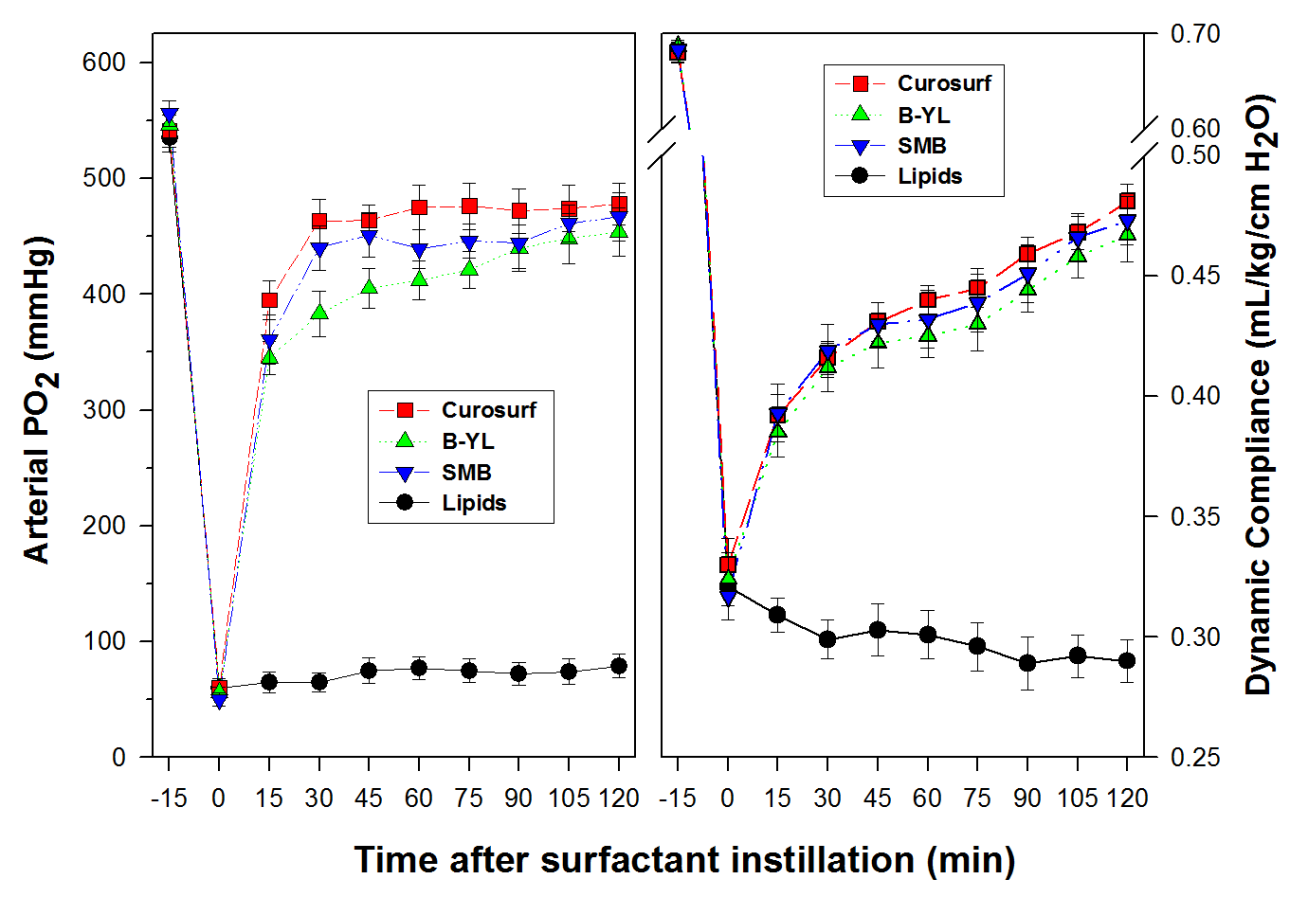
Arterial oxygenation and dynamic lung compliance in lavaged, surfactant-deficient, ventilated young adult rabbits treated with surfactant. Oxygenation (arterial PO _2_ in mmHg) and dynamic lung compliance (mL/kg/cm H _2_O) of 3 mole% B-YL (n=9) and SMB (n=8) in surfactant lipids were compared with a clinical surfactant (Curosurf®) (n=8) or lipids alone (DPPC:POPC:POPG 5:3:2 mole:mole:mole) (n=8). Surfactant (100 mg/kg) was administered intratracheally as a bolus at time 0. Values are mean ± SEM. Differences in oxygenation and dynamic compliance between B-YL, SMB and Curosurf surfactants were not statistically significant, except for slightly reduced PaO _2_ values for B-YL surfactant compared to Curosurf®) (p<0.03), but not to SMB, during the first 75 min post-instillation. Differences between B-YL, SMB and Curosurf®) surfactant with lipids alone were significant (p<0.001).

Animal experiments directly examined the *in vivo* pulmonary activity of B-YL surfactant when instilled intratracheally in ventilated young adult rabbits with surfactant deficiency and impaired lung function induced by repeated saline lung lavages ( Figure 6). Surfactant was administered by intratracheal instillation after the PaO _2_ was reduced to stable levels <100 mmHg when breathing 100% oxygen. For this timeframe of study, this model reflects a relatively pure state of surfactant deficiency in animals with mature lungs. Rabbits receiving B-YL, SMB and Curosurf® surfactant had significantly improved arterial oxygenation over the 2 h period of study post-instillation compared to control rabbits instilled with surfactant lipids alone ( Figure 6, p<0.001). Dynamic lung compliance also significantly improved over the same period of post-instillation study in rabbits treated with B-YL and SMB surfactants and Curosurf® compared to lipid-only controls ( Figure 6). During the first 75 min after surfactant administration, mean PaO _2_ values of the B-YL group were slightly lower than in the Curosurf® group (p<0.03), but were not different from those in the SMB group. Thereafter, mean PaO _2_ values were similar among the three active surfactant preparations. Dynamic lung compliance of B-YL and SMB surfactants and Curosurf® was not statistically significant different throughout the study period. These data indicate that the modifications in the SP-B peptide mimic B-YL did not lead to a loss in *in vivo* surface activity, despite a difference in initial kinetics compared to Curosurf®.

## Discussion

The basic premise tested here was whether the ‘sulfur-free’ B-YL peptide, a 41-residue Super Mini-B (SMB) variant that has its four cysteine residues replaced by tyrosine and its two methionine residues replaced by leucine, would fold with the same α-helix hairpin conformation earlier shown by SMB and Mini-B to correlate with high *in vitro* and *in vivo* surfactant activities ^8^,^10^,^14^,^18^.

In the present studies, circular dichroism (CD) and FTIR spectroscopy of ‘B-YL’ in surfactant lipids showed secondary structures compatible with the peptide folding as an α-helix hairpin, similar to that of SMB in lipids ( Figure 2– Figure 4; Table 1). Moreover, captive bubble surfactometry indicated excellent surface activity for B-YL surfactant ( Figure 5), while also showing good oxygenation and dynamic compliance in lavaged, surfactant-deficient adult rabbits, an animal model of surfactant deficiency ( Figure 6). One possible explanation of this correlation between α-helix turn and surfactant activity in B-YL is that the disulfide bonds of the parent SMB have been replaced by a core of clustered Tyr that instead crosslinks the N- and C- helices through noncovalent interactions involving aromatic rings. In this context, it is worthwhile to compare these B-YL results with an earlier functional and NMR structural investigation of Tachyplesin I (TP-I) ^46^. TP-I is a 17-residue peptide that exhibits high antimicrobial activity and forms a β-hairpin (i.e., antiparallel β-sheet) stabilized by two disulfide cross-links. Interestingly, replacement of the disulfides with four tyrosines produced a TP-I analog (TP-1Y) with a stable β-hairpin conformation in solution and high antimicrobial activity, while the corresponding TP-I analog with four alanines (TP-IA) was unstructured in solution and inactive. Because of the proximity of the tyrosine side chains, Laederach *et al.* ^46^ proposed that the β-hairpin conformation and high antimicrobial activity of TP-IY were stabilized by aromatic ring stacking interactions (i.e., “π-stacking” interactions) ^27^,^29^. It is tempting to speculate that tyrosines may be playing a similar role in maintaining the α-helix hairpin structure and high surfactant activity of B-YL in lipid environments. To address remaining questions, we are planning long-time (≥ 1.0 µsec) production runs for all-atom MD simulations of B-YL to ensure that our final models are fully equilibrated in hydrated surfactant lipid bilayers. 

Our ‘sulfur-free’ B-YL mimic may prove to be superior to its parent SMB for treating premature infants with RDS for several reasons. First, B-YL is less expensive and faster to synthesize than SMB because it omits an oxidation step and is self-folding. Second, the substitution of Cys and Met residues with Tyr and Leu, respectively, may render the B-YL mimic less susceptible to inactivation by reactive oxygen species.

Lung immaturity and surfactant deficiency are the main cause of RDS in very preterm infants. Mechanical ventilation and exposure to high oxygen concentrations may lead to an inflammatory lung process resulting in bronchopulmonary dysplasia (BPD), a chronic lung disease of preterm infants. Use of antenatal steroids, administration of exogenous surfactant, and advanced modes of ventilation have shown only limited benefits in preventing BPD. In a neonatal rat model of hyperoxia-induced lung injury, we found that nebulized PPARγ agonist pioglitazone (PGZ) with B-YL surfactant accelerates lung maturation and prevents neonatal hyperoxia-induced lung injury more than with either modality alone, thereby potentially preventing BPD more effectively ^47^. These findings suggest a potential role for B-YL surfactant as a vehicle for intrapulmonary drug therapy.

## Conclusion

The ‘sulfur-free’ B-YL forms an amphipathic helix-hairpin in surfactant liposomes with high surface activity, and is functionally similar to its parent (SMB) and native SP-B. This self-folding peptide is easier to synthesize and may provide an extra edge over SMB in the treatment of respiratory failure in premature infants with RDS.

## Data availability

Raw data are available on OSF: http://doi.org/10.17605/OSF.IO/6295P ^48^

Data are available under the terms of the Creative Commons Zero "No rights reserved" data waiver (CC0 1.0 Public domain dedication).
