# Supplementary material for: A sulfur-free peptide mimic of surfactant protein B (B-YL) exhibits high in vitro and in vivo surface activities
Source: Gates Open Res. 2018 Jul 10;2:13. Originally published 2018 Feb 26. [Version 2] doi: 10.12688/gatesopenres.12799.2 (PMC6139377; doi:10.12688/gatesopenres.12799.2)
Supplement: Peer review history

# Peer review: Referee response for version 2

**DOI:** 10.21956/gatesopenres.13916.r26555

**Published:** 2018-07-27

**Authors:** Jesús Pérez-Gil

**Affiliations:** 1 Department of Biochemistry, Faculty of Biology and Research Institute Hospital 12 de Octubre, Complutense University, Madrid, Spain

All the questions raised have been properly addressed in my opinion.

I have read this submission. I believe that I have an appropriate level of expertise to confirm that it is of an acceptable scientific standard.

# Peer review: Referee response for version 2

**DOI:** 10.21956/gatesopenres.13916.r26554

**Published:** 2018-07-16

**Authors:** Valerie Booth

**Affiliations:** 1 Department of Biochemistry , Memorial University of Newfoundland, St. John's, NL, Canada

All concerns have been addressed.

I have read this submission. I believe that I have an appropriate level of expertise to confirm that it is of an acceptable scientific standard.

# Peer review: Referee response for version 1

**DOI:** 10.21956/gatesopenres.13861.r26281

**Published:** 2018-03-28

**Authors:** Jesús Pérez-Gil

**Affiliations:** 1 Department of Biochemistry, Faculty of Biology and Research Institute Hospital 12 de Octubre, Complutense University, Madrid, Spain

This study summarizes data on the design, synthesis, structure, lipid-protein interactions and surface activity -in vitro and in vivo- of a synthetic peptide analog that mimics certain functional segments of pulmonary surfactant protein SP-B. The team responsible has a large expertise in the design and optimization of this type of peptide mimics of surfactant proteins, and the current work is a natural and smart extension of previous work.

 In the current study, an analog to a well-know synthetic surrogate of SP-B, the so-called “mini-B”, has been produced and analyzed in detail. This ‘B-YL’ peptide is a mini-B version in which cysteines forming disulphide bonds, in both wild-type SP-B ad mini-B, have been substituted by tyrosines. The rational is that tyrosines could also contribute to stabilize peptide-peptide interactions via pi-stacking and so yielding the typical alpha-helical hairpin bridging N-terminal and C-terminal helical segments of SP-B together. The substitution of Cys by Tyr would on the other hand supposedly render a peptide surrogate with lower propensity to oxidation, according to the authors, thanks to the elimination of cysteine residues. The authors essentially conclude that the B-YL analog is entirely comparable, structurally and functionally, to the mini-B construct, and therefore that it would be equally useful to produce synthetic surfactants for therapeutic applications.

 In general term, the experiments have been properly carried out, and they provide extensive proofs that the peptide behaves “in general terms” as it was expected. In this sense, it is interesting to see how the substitution of two disulphides by tyrosine clustering could also serve as a basis to build a membrane active peptide fold such as the one of miniB. This is certainly an advantage from the point of view of its synthesis and production. It would be for instance a large advantage to produce inexpensive large amounts of this type of peptides by overexpression in bacteria, where formation of disulphides would certainly be a limitation.

 What I am not so sure is whether this peptide would actually be less susceptible to oxidation, and whether this peptide is actually a good mimic of SP-B, or at least as good as miniB.

 As mentioned by the authors, one of the targets of oxidation-promoted inactivation of surfactant proteins are the aromatic residues. Actually, oxidation of the single tryptophan of SP-B (as shown by the group of Possmayer), located at its N-terminal helical segment, is associated with complete inactivation of the protein. It had been previously demonstrated (see for instance Ryan et al.(2005) ^1^ or Serrano et al.(2006) ^2^) that this tryptophan is essential at the N-terminal end of SP-B, because it takes part of a highly hydrophobic-at-interface segment which defines very high affinity for insertion into amphipathic environments such as the air-liquid interface. Now this tryptophan has been substituted in the whole peptide by 4 tyrosines. Tyrosines may maintain a high affinity for the interface, but at the cost of being also highly susceptible to oxidation. The authors could have shown if it is true that exposure of B-YL to oxidant reactions preserve its activity better than in the case of mini-B.

 Another major problem in my opinion of the substitution of disulphides by aromatic residues is that it may change dramatically the way the peptide perturb surfactant membranes and films, which is crucial for a good surface activity. Disulphides surely impose a very strong constraint to the way the two helical segments orientate into the membrane. As a matter of fact, the tilting of the amphipathic N-terminal and C-terminal helical segments of SP-B, which is crucial for perturbing lipid packing to the point of facilitating the transfer of surface active lipid molecules between different bilayers or between the bilayers and the interfacial film, may be subtly different in the case of miniB, and even more in the B-YL analog. I may agree that tyrosine clustering could roughly join the C-terminal and N-terminal segments together, but they would do that in a much more flexible, dynamic and fluctuating way than the disulphides, and that may reduce the efficiency for the protein to induce membrane perturbation. This could be the reason why the maximum surface tension during cycling of lipid-peptide films at the CBS is higher in the case of B-YL than in mini-B, and much more than in the case of Curosurf containing true SP-B, where the full protein structure may impose a much more defined geometry to the N-terminal/C-terminal helical cluster. The limited MD simulations in the presence of lipids also seem to show differences in tilting of the helical segments with respect to the lipid surface. The authors have neglected these differences, but it would be important to show if they do really exist and whether they are reproducible.

 Other problem related with the final geometry of the lipid-protein complexes comes from the high hydrophobicity-at interface of the aromatic residues. The introduction of the 4 tyrosines may force a orientation of the mini-B construct which is much more anchored to the surface of the membrane, where aromatic residues tend to partitition, than it is the case for the tyrosine-free miniB analog.

 The authors may argue that the MD simulations do not reflect substantial differences in this respect, but a 500 nsec simulation may or may not be enough to get the fully equilibrated state. Have the authors data to evaluate whether longer simulation times could reflect further differences?

 There is an additional important issue that may arise from the substitution of disulphides by tyrosines that in my opinion has not been properly addressed. If tyrosine clustering is enough to sustain intramolecular peptide-peptide interactions stabilizing the miniB hairpin, it may also sustain intermolecular peptide-peptide interactions, which could end in peptide aggregation and clustering, potentially reducing lipid dispersion and surface activity. The simulations run do not contain multiple copies of the peptide, which could assess whether intramolecular interactions are or not preferred over intermolecular interactions, provided that long enough simulation times are applied. This problem does not exist, obviously, when the intramolecular interactions are maintained through covalent disulphide bonds.

 Other questions that would require some discussion include:

 The use of surfactant therapy on therapies to treat ALI and ARDS is under strong debate. It is doubtful that a simple synthetic surfactant such as those containing mini-B-like peptides as surrogates of SP-B would be ever useful to treat ARDS patients.

 The way captive bubble experiments have been performed has been poorly explained and produce some confusion. In page 6, it is stated that CBS has been operated at “physiological cycling rate”. However, in page 14, it is stated that “Surface activity of … were consistently and equally low during quasi-static cycling…” What has been the compression-expansion cycling rate used? There is also some confusion with respect to the concentration at which the different surfactants have been tested in the CBS.  In page 6, it is stated that “We routinely analyze surfactant preparations at an average surfactant lipid concentration of 25 microgram/mL”. Later it is stated that “We used a B-YL surfactant mixture consisting on… with a concentration of 35 mg/mL”. Could you, please, clarify what is the lipid concentration used to obtain each of the CBS isotherms? How this concentration is applied (in the bulk hypophase in which the bubble is formed, or directly at the air-water interface)?

 The results of MD simulations have been taken as a proof that the cys-by-tyr substitutions can also sustain the intramolecular peptide-peptide interactions defining the formation of the miniB-like helical hairpin in surfactant lipids. The question is what were the initial points of at which those simulations were started? Where the aromatic sidechains already clustered or where the peptides initially embedded into the membranes in an extended conformation, to see whether the propensity for the hairpin to be formed could be entirely sustained by the affinity of tyrosines to pile? This second type of experiments could be crucial. Even better if the simulation cell could contain not one but several copies of the peptides.

 The experiments in vivo seems to indicate that the B-YL analog is less active than miniB or Curosurf to produce good oxygenation indexes, at least at the shortest times. The authors state that there is no statistical difference between the activity in vivo of the two different analogs. Have they tested statistical significance of the means only at the end times or have they checked whether differences at shorter times could indicate that there may be a significant difference in kinetics (which can also functionally important)?

 In summary, I think the study can be of interest for the scientists in the field, and may open alternatives in the design of other peptides or proteins potentially mimicking the structure-function determinants of surfactant proteins. However, instead of merely summarizing the main parallelisms found, the Discussion should be completed by addressing all the questions raised in this report, which could add substantially to the structural and functional features that may be important to test when designing future new peptide surfactant additives for therapeutic purposes.

I have read this submission. I believe that I have an appropriate level of expertise to confirm that it is of an acceptable scientific standard, however I have significant reservations, as outlined above.

## References

[1] RyanMAQiXSerranoAGIkegamiMPerez-GilJJohanssonJWeaverTE: Mapping and analysis of the lytic and fusogenic domains of surfactant protein B. *Biochemistry*.2005;44(3) : 10.1021/bi0485575 861-72 10.1021/bi0485575 15654742

[2] SerranoAGRyanMWeaverTEPérez-GilJ: Critical structure-function determinants within the N-terminal region of pulmonary surfactant protein SP-B. *Biophys J*.2006;90(1) : 10.1529/biophysj.105.073403 238-49 10.1529/biophysj.105.073403 16214863PMC1367022

# Response

**Published:** 2018-07-04

**Authors:** Frans Walther

**Affiliations:** LA BioMed at Harbor-UCLA Medical Center, USA

**Response to Reviewer Dr. Pérez-Gil:**

1. What I am not so sure is whether this peptide would actually be less susceptible to oxidation, and whether this peptide is actually a good mimic of SP-B, or at least as good as miniB.

As mentioned by the authors, one of the targets of oxidation-promoted inactivation of surfactant proteins are the aromatic residues. Actually, oxidation of the single tryptophan of SP-B (as shown by the group of Possmayer), located at its N-terminal helical segment, is associated with complete inactivation of the protein. It had been previously demonstrated (see for instance Ryan et al.(2005)  or Serrano et al.(2006) ) that this tryptophan is essential at the N-terminal end of SP-B, because it takes part of a highly hydrophobic-at-interface segment which defines very high affinity for insertion into amphipathic environments such as the air-liquid interface. Now this tryptophan has been substituted in the whole peptide by 4 tyrosines. Tyrosines may maintain a high affinity for the interface, but at the cost of being also highly susceptible to oxidation. The authors could have shown if it is true that exposure of B-YL to oxidant reactions preserve its activity better than in the case of mini-B.

Response: As suggested by the other reviewer, Dr. Valerie Booth, we decided to remove all claims of “oxidation-resistance” in the title, abstract, conclusion and other parts of the text. The B-YL peptide is somewhat less sensitive to oxidation by Leu substitution for Met and storage for one year showed no Tyrosine or Tryptophan oxidation by mass spec. B-YL is actually as good a mimic of SP-B as Super Mini-B at captive bubble surfactometry and in the lavaged, surfactant-deficient rabbits.

2.  Another major problem in my opinion of the substitution of disulphides by aromatic residues is that it may change dramatically the way the peptide perturb surfactant membranes and films, which is crucial for a good surface activity. Disulphides surely impose a very strong constraint to the way the two helical segments orientate into the membrane. As a matter of fact, the tilting of the amphipathic N-terminal and C-terminal helical segments of SP-B, which is crucial for perturbing lipid packing to the point of facilitating the transfer of surface active lipid molecules between different bilayers or between the bilayers and the interfacial film, may be subtly different in the case of miniB, and even more in the B-YL analog. I may agree that tyrosine clustering could roughly join the C-terminal and N-terminal segments together, but they would do that in a much more flexible, dynamic and fluctuating way than the disulphides, and that may reduce the efficiency for the protein to induce membrane perturbation. This could be the reason why the maximum surface tension during cycling of lipid-peptide films at the CBS is higher in the case of B-YL than in mini-B, and much more than in the case of Curosurf® containing true SP-B, where the full protein structure may impose a much more defined geometry to the N-terminal/C-terminal helical cluster. The limited MD simulations in the presence of lipids also seem to show differences in tilting of the helical segments with respect to the lipid surface. The authors have neglected these differences, but it would be important to show if they do really exist and whether they are reproducible.

Other problem related with the final geometry of the lipid-protein complexes comes from the high hydrophobicity-at interface of the aromatic residues. The introduction of the 4 tyrosines may force a orientation of the mini-B construct which is much more anchored to the surface of the membrane, where aromatic residues tend to partitition, than it is the case for the tyrosine-free miniB analog.

Response: Simulations show no difference in surface orientation. However, we decided to remove the homology models, molecular dynamic (MD) simulation and Membrane Protein Explorer (MPEx) from the manuscript as modeling was only a secondary focus and we plan to publish a more extensive data set in a follow-up paper.

3. The authors may argue that the MD simulations do not reflect substantial differences in this respect, but a 500 nsec simulation may or may not be enough to get the fully equilibrated state. Have the authors data to evaluate whether longer simulation times could reflect further differences?

Response: We extended simulations to one microsecond. However, we have decided to remove the homology models, molecular dynamic (MD) simulation and Membrane Protein Explorer (MPEx) from the manuscript as modeling was only a secondary focus and we plan to publish a more extensive data set in a follow-up paper.

4.   There is an additional important issue that may arise from the substitution of disulphides by tyrosines that in my opinion has not been properly addressed. If tyrosine clustering is enough to sustain intramolecular peptide-peptide interactions stabilizing the miniB hairpin, it may also sustain intermolecular peptide-peptide interactions, which could end in peptide aggregation and clustering, potentially reducing lipid dispersion and surface activity. The simulations run do not contain multiple copies of the peptide, which could assess whether intramolecular interactions are or not preferred over intermolecular interactions, provided that long enough simulation times are applied. This problem does not exist, obviously, when the intramolecular interactions are maintained through covalent disulphide bonds.

Response: Insertion sequence keeps this from happening. However, we decided to remove the homology models, molecular dynamic (MD) simulation and Membrane Protein Explorer (MPEx) paragraphs from the manuscript as modeling was only a secondary focus and plan to publish a more extensive data set in a follow-up paper.

5. The use of surfactant therapy on therapies to treat ALI and ARDS is under strong debate. It is doubtful that a simple synthetic surfactant such as those containing mini-B-like peptides as surrogates of SP-B would be ever useful to treat ARDS patients.

Response: We agree with the reviewer and removed the statements about surfactant therapy for ALI/ARDS from the abstract, the last paragraph of page 3, discussion and conclusion.

6. The way captive bubble experiments have been performed has been poorly explained and produce some confusion. In page 6, it is stated that CBS has been operated at “physiological cycling rate”. However, in page 14, it is stated that “Surface activity of … were consistently and equally low during quasi-static cycling…” What has been the compression-expansion cycling rate used? There is also some confusion with respect to the concentration at which the different surfactants have been tested in the CBS.  In page 6, it is stated that “We routinely analyze surfactant preparations at an average surfactant lipid concentration of 25 microgram/mL”. Later it is stated that “We used a B-YL surfactant mixture consisting on… with a concentration of 35 mg/mL”. Could you, please, clarify what is the lipid concentration used to obtain each of the CBS isotherms? How this concentration is applied (in the bulk hypophase in which the bubble is formed, or directly at the air-water interface)?

Response: We actually do both quasi-static and dynamic cycling on the CBS. Quasi-static compression and expansion of the air bubble is performed in discrete steps at a rate of 5% of the bubble volume every 10 sec with continuous video recording of the bubble shape. Dynamic compression and expansion cycling is performed between 10 and 110% of the original bubble area at a cycling rate of 20 cycles/min. Both modalities show extreme flattening of the bubble in active surfactant preparations. In figure 9 we present the data from quasi-static cycling. We routinely analyze surfactant preparations at an average surfactant lipid concentration of 25 µg/mL in the bubble chamber, but as Curosurf® is more concentrated than synthetic surfactant, we applied 1 µL of synthetic surfactant at 35 mg/mL and 0.5 µL of Curosurf® at 80 mg/mL to the bubble chamber (1.5 mL volume). After introducing surfactant and the air bubble into the CBS chamber, both move upwards to the interface with the agarose plug. This information was added to the methods section.

7. The results of MD simulations have been taken as a proof that the cys-by-tyr substitutions can also sustain the intramolecular peptide-peptide interactions defining the formation of the miniB-like helical hairpin in surfactant lipids. The question is what were the initial points of at which those simulations were started? Where the aromatic sidechains already clustered or where the peptides initially embedded into the membranes in an extended conformation, to see whether the propensity for the hairpin to be formed could be entirely sustained by the affinity of tyrosines to pile? This second type of experiments could be crucial. Even better if the simulation cell could contain not one but several copies of the peptides.

Response: See above, we decided to remove the homology models, molecular dynamic (MD) simulation and Membrane Protein Explorer (MPEx) from the manuscript as modeling was only a secondary focus and we plan to publish a more extensive data set in a follow-up paper.

8. The experiments in vivo seems to indicate that the B-YL analog is less active than miniB or Curosurf to produce good oxygenation indexes, at least at the shortest times. The authors state that there is no statistical difference between the activity in vivo of the two different analogs. Have they tested statistical significance of the means only at the end times or have they checked whether differences at shorter times could indicate that there may be a significant difference in kinetics (which can also functionally important)?

Response: In response to the reviewer, we calculated the statistical significance of differences in means at every 15 min time-point after surfactant instillation and found no significant differences in dynamic compliance for the 3 surface-active surfactant preparations throughout the experiments. During the first 75 min after surfactant administration, mean oxygenation values of the B-YL surfactant group were slightly lower than in the Curosurf® group  (p<0.03), but not different from those in the Super Mini-B (SMB) group, suggesting initial differences in kinetics. Thereafter oxygenation was similar in B-YL, SMB and Curosurf® surfactants. This info was added to the results section.

# Peer review: Referee response for version 1

**DOI:** 10.21956/gatesopenres.13861.r26299

**Published:** 2018-03-20

**Authors:** Valerie Booth

**Affiliations:** 1 Department of Biochemistry , Memorial University of Newfoundland, St. John's, NL, Canada

Walther, Gupta, Gordon and Waring present studies of a peptide called “B-YL” which they propose may be of utility as a component of exogenous lung surfactant replacement therapy for conditions such as ARDS and ALI. In these conditions, the lung environment is “hostile” to lung surfactant proteins and a variety of inactivating components including reactive oxygen species are present. The experimental work is comprehensive and complementary, as well as expertly done and clearly described (with a few exceptions as detailed below). I appreciate the effort to include the *in vitro* and *in vivo* functional work, as well as the structural studies. I also believe that the general thrust of the work – creating peptide components that can act in place of full length lung surfactant proteins – is very worthwhile.

 However, I do have trouble with the premise for the paper – that removing the cysteines and methionines will make the peptide “oxidation-resistant”. The authors present no justification that cysteines are a target of ROS damage. In the lung surfactant literature, it’s been shown that tryptophan 9 is the main target of oxidation, with methionine residues as a secondary target ^1^. The peptide B-YL leaves this critical tryptophan unchanged and so may well be just as susceptible to damage as the parent peptide, super Mini-B. Furthermore, it’s been proposed that one of the roles of methionines may be to protect the tryptophan by competing for ROS. So, removing the methionines may actually make the protein MORE susceptible to damage. Thus, I think the options are to either back up the assertion that the new peptide is “oxidation-resistant” with convincing experiments or references OR to remove all claims of “oxidation-resistant” from the title, abstract, conclusion and other parts of the text. I think the work stands up perfectly well without the oxidation-resistant angle, because as the authors point out, the removal of the cysteines makes it more feasible to scale up production to the scale needed for clinical work.

 Here are the rest of my comments, roughly in the same order as the manuscript:

1. Page 3 bottom left. Could you add a sentence to describe what an alpha-helix hairpin is. It’s referred to several times later on so it would be good to start with a clear picture of what it is.
2. Page 3, right hand column, 15 lines from the bottom reads “Trp-9 plays a role in the diminished surface activity possibly due to fraying of the N-terminal alpha-helix 24,25”. Reference 25 actually suggests the activity may be changed because the oxidizing Trp to kynurenine causes the peptide to change its depth within the lipid layer.
3. Page 3, right hand column, 1 sentence of the last paragraph. I’m not sure we can say that “ALI/ARDS… REQUIRES the use of exogenous surfactants” since I’m not sure that it’s ever been convincingly shown that exogenous surfactant treatment substantially helps patients with ALI/ARDS – can you re-phrase or back-up this statement.
4. Page 3, last sentence. It would be good to explain why tyrosine (instead of some other amino acid) was chosen to replace the Cys and Met.
5. Methods paragraph 2 and throughout. It is very (very!) confusing that Super Mini-B is referred to throughout as “oxidized” SMB. The “oxidation” is just the normal formation of disulfide bonds and since the paper is supposedly about making a “oxidation-resistant” version of SMB the two meanings are bound to be confounded by readers. I would just call it “SMB with disulfides” or something else along those lines.
6. Page 5, last paragraph. Instead of referring to a “recent homology modeling program” and then naming the actual program 5 lines later, I would put the name of the program right into this first sentence.
7. Page 5, last paragraph – which pdb files went into the homology model? I think it’s important to know this as there’s not that much sequence homology between SP-B and known structures so whichever ones I-TASSER is choosing will have a big influence on how the structures look.
8. Page 5 under the MD section… what was the initial position of the peptide within the bilayer and how did it change during the simulation? Note that Khatami et al. ^2^ found that the secondary structure of full length SP-B in MD simulations varied a lot with the position with respect to the bilayer. This is probably less important for the smaller peptide, but is still worth mentioning the initial position and the rationale for it.
9. Page 6 under Captive bubble surfactometry. I’m guessing 3% of B-YL peptide is by mole? Can you add this info (here and later on) so we’re clear?
10. Page 7 top left. At some point (not necessarily here) it would be good to compare the dose of surfactant (in mg/kg body weight) to what is given to preemies. Also could you comment on why the dose is lower for Curosurf than for SMB?
11. Page 7. First paragraph under results and Table 1. I’m not sure its appropriate to quote “Beta-sheet” content since I don’t think you are proposing there is an actual sheet involved. Could you choose something more appropriate, like “beta-strand” or “beta-hairpin” or just “beta-structure”.
12. Figure 3. Since estimating secondary structure is so dependent on protein concentration, it would be good to know what method was used to determine the peptide concentration (desalted dry weight, UV, colorimeteric assay ?).
13. Page 10, and later. The “core of hydrophobic residues” should be compared to that of Mini-B’s experimental structure. How much of this core is preserved in your predictions for the SMB and B-YL structures?
14. Page 10, bottom left and elsewhere. Can you replace all instances of “largely agrees with” with something more quantitative – like backbone RMSD.
15. Page 11, left hand column. In addition to the pi stacking interactions, I’m wondering what the hydroxyl on the tyrosines are doing – are they finding anything to hydrogen bond to? What?
16. Page 11, 2 sentence of the 2 paragraph: as for 14 above, please replace “generally in good agreement” with something more precise.
17. Page 11 – How do the starting and final structures and hydrophobic core compare to the experimental structure for Mini-B in SDS micelles?
18. Page 14 – right hand side, please replace “good overlap” and “remarkably analogous” with something more precise like RMSD.

I have read this submission. I believe that I have an appropriate level of expertise to state that I do not consider it to be of an acceptable scientific standard, for reasons outlined above.

## References

[1] ManzanaresDRodriguez-CapoteKLiuSHainesTRamosYZhaoLDoherty-KirbyALajoieGPossmayerF: Modification of tryptophan and methionine residues is implicated in the oxidative inactivation of surfactant protein B. *Biochemistry*.2007;46(18) : 10.1021/bi062304p 5604-15 10.1021/bi062304p 17425286

[2] KhatamiMHSaika-VoivodIBoothV: All-atom molecular dynamics simulations of lung surfactant protein B: Structural features of SP-B promote lipid reorganization. *Biochim Biophys Acta*.1858(12) : 10.1016/j.bbamem.2016.09.018 3082-3092 10.1016/j.bbamem.2016.09.018 27671054

# Response

**Published:** 2018-07-04

**Authors:** Frans Walther

**Affiliations:** LA BioMed at Harbor-UCLA Medical Center, USA

**Response to Reviewer Dr. Valerie Booth:**

General response

Suggestion to remove all claims of “oxidation-resistance” from the title, abstract, conclusion, and other parts of the text.

Response: We removed all claims of “oxidation-resistance” from the title, abstract, conclusion, and text. As B-YL peptide is sulfur-free, the title was changed to: “A sulfur-free peptide mimic of surfactant protein B (B-YL) exhibits high in vitro and in vivo surface activities”.

**Specific comments:**

1. Page 3, bottom left. Could you add a sentence to describe what an alpha-helix hairpin is. It’s referred to several times later on so it would be good to start with a clear picture of what it is.

Response: We have added to Page 3 bottom left a definition of an alpha helix hairpin structure as well as provided a literature reference for it: “SMB incorporates the N-terminal α-helix (~residues 8-25) and C-terminal α-helix (~residues 63-78) of native SP-B as a single linear peptide (Figure 1A), joined together with a customized turn to form a α-helix hairpin (a-helix/turn/a-helix, ata) [Fezoui Y et al., 1994].“

2. Page 3, right hand column, 15 lines from the bottom reads “Trp-9 plays a role in diminished surface activity possibly due to fraying of the N-terminal alpha helix 24-25”. Reference 25 actually suggest the activity may be changed because the oxidizing Trp to kynurenine causes the peptide to change its depth within the lipid layer.

Response: We have added here that this is in the full-length protein and in addition we have incorporated mass spec findings in the results section that indicate that the B-YL peptide does not undergo oxidation of its aromatic amino acids when stored in surfactant lipids for one year:

“Trp-9 can play a role in diminished surface activity in the full length native protein possibly due to fraying of the N-terminal alpha helix 24-25”.

“Mass spectral analysis of the B-YL peptide indicated there was no change in the molecular weight due to oxidation of tyrosines or tryptophan for one year when formulated with surfactant lipids.”

3. Page 3, right hand column, 1 sentence of the last paragraph. I’m not sure we can say that “ALI/ARDS… REQUIRES the use of exogenous surfactants” since I’m not sure that it’s ever been convincingly shown that exogenous surfactant treatment substantially helps patients with ALI/ARDS – can you re-phrase or back-up this statement.

Response: We agree with the reviewer and removed this sentence and “surfactant dysfunction in ALI/ARDS” in the next sentence.

4. Page 3, last sentence. It would be good to explain why tyrosine (instead of some other amino acid) was chosen to replace the Cys and Met.

Response: We have added an additional sentence (line 2, page 4) to explain our rational in the selection of tyrosine and leu for cysteine and methionine. We opted for leucine rather than the commonly used norleucine because we felt that the use of non-natural amino acids may cause longer term metabolic problems *in vivo*.

“Tyrosine was substituted for cysteine because of its aromatic ring interactions that emulate disulfide formation [55, 56, 57] and methionine, that is easily oxidized, was replaced by leucine based on its similar molecular structure and polarity [44].”

5. Methods paragraph 2 and throughout. It is very (very!) confusing that Super Mini-B is referred to throughout as “oxidized” SMB. The “oxidation” is just the normal formation of disulfide bonds and since the paper is supposedly about making a “oxidation-resistant” version of SMB the two meanings are bound to be confounded by readers. I would just call it “SMB with disulfides” or something else along those lines.

Response: We have modified the text using “SMB” instead of “oxidized SMB” in accordance with the reviewer’s comments, so that there is no confusion with oxidation of the peptide per se.

6. Page 5, last paragraph. Instead of referring to a “recent homology modeling program” and then naming the actual program 5 lines later, I would put the name of the program right into this first sentence.

Response: We decided to remove the homology models, molecular dynamic (MD) simulation and Membrane Protein Explorer (MPEx) from the manuscript as modeling was only a secondary focus and we plan to publish a more extensive data set in a follow-up paper.

7. Page 5, last paragraph – which pdb files went into the homology model? I think it’s important to know this as there’s not that much sequence homology between SP-B and known structures so whichever ones I-TASSER is choosing will have a big influence on how the structures look.

Response: Please see our response to reviewer’s comment #6.

8. Page 5 under the MD section… what was the initial position of the peptide within the bilayer and how did it change during the simulation? Note that Khatami et al.  found that the secondary structure of full length SP-B in MD simulations varied a lot with the position with respect to the bilayer. This is probably less important for the smaller peptide, but is still worth mentioning the initial position and the rationale for it.

Response: Please see our response to reviewer’s comment #6.

9. Page 6 under Captive bubble surfactometry. I’m guessing 3% of B-YL peptide is by mole? Can you add this info (here and later on) so we’re clear?

Response: We used 3 mole % of B-YL or SMB peptide. We have clarified the concentration by including a reference [17] detailing the formulation used for SMB and B-YL peptides.

10. Page 7 top left. At some point (not necessarily here) it would be good to compare the dose of surfactant (in mg/kg body weight) to what is given to preemies. Also could you comment on why the dose is lower for Curosurf than for SMB?

Response: All animals received 100 mg lipids/kg bodyweight of one of the 4 surfactant preparations. The clinical surfactant Curosurf® is formulated by the manufacturer at 80 mg/mL and SMB and B-YL surfactant and lipids alone were formulated at 35 mg/mL, so Curosurf® was dosed at 1.25 mL/kg and the synthetic surfactants at 2.9 mL/kg. Clinical surfactant is routinely dosed at 100 mg/kg in premature infants with RDS, so the dosages used here were similar to those used clinically. This info was added to the methods on page 7.

11. Page 7. First paragraph under results and Table 1. I’m not sure its appropriate to quote “Beta-sheet” content since I don’t think you are proposing there is an actual sheet involved. Could you choose something more appropriate, like “beta-strand” or “beta-hairpin” or just “beta-structure”.

Response: We have replaced “Beta-sheet” with “beta-structure”.

12. Figure 3. Since estimating secondary structure is so dependent on protein concentration, it would be good to know what method was used to determine the peptide concentration (desalted dry weight, UV, colorimeteric assay ?).

Response: We have added the following description and reference with regard to peptide quantitation: “Peptides were routinely quantitated using UV absorbance based on the assay procedure developed by Anthis and Clore (Protein Science, 2013)”.  

13. Page 10, and later. The “core of hydrophobic residues” should be compared to that of Mini-B’s experimental structure. How much of this core is preserved in your predictions for the SMB and B-YL structures?

Response: Please see our response to reviewer’s comment #6.

14. Page 10, bottom left and elsewhere. Can you replace all instances of “largely agrees with” with something more quantitative – like backbone RMSD.

Response: Please see our response to reviewer’s comment #6.

15. Page 11, left hand column. In addition to the pi stacking interactions, I’m wondering what the hydroxyl on the tyrosines are doing – are they finding anything to hydrogen bond to? What?

Response: Please see our response to reviewer’s comment #6.

16. Page 11, 2 sentence of the 2 paragraph: as for 14 above, please replace “generally in good agreement” with something more precise.

Response: Please see our response to reviewer’s comment #6.

17. Page 11 – How do the starting and final structures and hydrophobic core compare to the experimental structure for Mini-B in SDS micelles?

Response: Please see our response to reviewer’s comment #6.

18. Page 14 – right hand side, please replace “good overlap” and “remarkably analogous” with something more precise like RMSD.

Response: Please see our response to reviewer’s comment #6.
